# An enhanced energy management framework based on artificial gorilla troops for optimal operation of grid-connected multi-nanogrids

**DOI:** 10.1038/s41598-026-45884-5

**Published:** 2026-04-18

**Authors:** W. T. Elsayed, A. Abdulnabi, A. A. Ali, E. M. Saied, Mohamed Selmy

**Affiliations:** 1 Department of Electrical Engineering, Faculty of Engineering at Shoubra, Benha University, Banha, Egypt; 2 Department of Electrical Engineering, Faculty of Engineering, Helwan University, Cairo, Egypt

**Keywords:** Energy management system (EMS), Nanogrids (NGs), Artificial gorilla troops optimizer (AGTO), Demand side management (DSM), Energy science and technology, Engineering, Mathematics and computing

## Abstract

The deployment of distributed energy resources (DERs) into power systems significantly improves their efficiency and reliability. Nanogrids (NGs), as small-scale systems that integrate DERs at the building level, require effective energy management to achieve optimal economic operation. This manuscript proposes an enhanced energy management system (EMS) for grid-connected NGs that combines day-ahead and real-time scheduling to minimize daily energy cost while maintaining the balance between power supply and demand. The day-ahead scheduling consists of two stages: first, applying demand-side management (DSM) using the load shifting approach with the day-ahead pricing curve; and second, determining the optimal powers of the DERs within the NGs. These resources are dynamically adjusted in real time to account for uncertainties in renewable generation, grid electricity prices, and load variations. Since energy scheduling is a complex, nonlinear optimization problem with multiple constraints, a recently developed metaheuristic technique, the Artificial Gorilla Troops Optimizer (AGTO), is proposed to obtain efficient solutions, and it is compared with different techniques such as the Honey Badger Algorithm (HBA), Aquila Optimizer (AO), and Particle Swarm Optimization (PSO). Simulation results show that the proposed AGTO-based EMS for grid-connected NGs achieves superior cost efficiency, saving approximately 15.83% compared to other approaches when determining the optimal setpoints of diesel generators and batteries, considering DSM in day-ahead scheduling.

The widespread integration of nanogrids (NGs) into the power system offers numerous benefits, including enhanced grid reliability, efficient rural electrification that avoids the costs and losses of extended transmission lines, and reduced carbon emissions^1–3^. A nanogrid (NG) is defined as a localized energy system including distributed energy resources (DERs) such as solar photovoltaics, wind turbines, batteries, and diesel generators with a total capacity of no more than 100 kW to feed a small building application or a single house. It has two operational modes: grid-connected operation for energy exchange and islanded operation for autonomous power supply during disconnection events^3^. NGs are divided into hybrid, DC, and AC architectures, determined by the bus configuration linking sources and loads^3,4^. A cluster of NGs is formed when some NGs are connected, allowing individual systems to share resources and enhance reliability under both islanded and grid-connected modes. Individual NGs can trade surplus electricity to balance local loads. Multi-connected nanogrids (MNGs) can be connected to a common DC bus to remove the complexities inherent in AC synchronization^5,6^. Regardless of configuration, an enhanced energy management system (EMS) must be deployed on both the supply and demand sides of NGs to minimize energy costs, optimize DERs operation, and sustain power quality^7–9^. This study focuses on managing the operation of grid-connected multi-nanogrids.

Since wind speed, solar intensity, grid prices, and load fluctuate continuously, the energy management system of NGs must effectively address these uncertainties to ensure the optimal operation of multi-nanogrid systems. Previous studies on energy management of NGs have primarily focused on enhancing power reliability^10^, reducing operational costs^11,12^, solving demand-side management problems^9,13^, integrating battery energy storage systems^14,15^, and implementing price-based scheduling strategies^16,17^. In this context, a comprehensive review of optimization-based energy management strategies is essential to critically examine their methodological frameworks, modeling techniques, and limitations in handling uncertainty.

In^6^, an energy management system for a low-voltage DC microgrid was formulated using a detailed mixed-integer nonlinear programming (MINLP) model solved via the global optimization solver BARON to minimize operating cost and emissions. In^12^, the authors proposed a multi-objective optimization framework for integrated energy systems that combines STA with fuzzy TOPSIS for decision-making to balance cost, emissions, and reliability. In^18^, a mixed-integer programming framework for unit commitment in a microgrid integrating incentive-based demand response and a battery energy storage system to enhance economic operation was proposed. In^19^, an uncertainty-based dynamic economic dispatch framework was proposed that considers diverse load demand and wind power profiles using a novel hybrid optimization algorithm to improve economic performance under variability conditions. In^20^, a decentralized control architecture for clustered DC nanogrids was proposed, aimed at enhancing voltage stability and power sharing in rural electrification applications. However, the aforementioned studies did not consider demand-side management strategies within their proposed frameworks.

In^4^, a pseudo-hierarchical DC nanogrid architecture integrating vehicle-to-grid (V2G) technology to enable both autonomous coordination and regulated power dispatch was proposed to enhance energy efficiency and cost-effectiveness. In^7^, the optimal supply-side and demand-side management strategies was developed for residential buildings using PSO to improve overall energy efficiency. In^21^, the demand-side management strategies in a hybrid rooftop photovoltaic-integrated smart nanogrid was investigated to improve energy utilization and reduce operational costs. In^22^, an EMS was proposed utilizing advanced evolutionary algorithms, considering the uncertainties in load and weather conditions during day-ahead. In^23^, an energy management system was proposed for multiple interconnected microgrids that operates under both grid-connected and autonomous modes, incorporating load management to improve operational efficiency. In^24^, a cost-effective grid-connected microgrid operational model integrating PHEVs and DSM using a bi-level differential evolution-based optimization approach was developed. In^25^, a comparative study of advanced evolutionary algorithms was conducted to optimize microgrid performance under dynamic pricing conditions, aiming to improve cost efficiency and operational reliability. In^26^, the demand-side management approach was proposed to solve environment-constrained economic dispatch in microgrid systems using a hybrid MGWOS-CACSA algorithm, aiming to improve both cost efficiency and environmental performance. In^27^, a multi-agent AI architecture for coordinating modular NGs was proposed. Nevertheless, some of the reviewed studies did not include uncertainty modeling in weather, load, and grid prices in their proposed frameworks^4,6,24,25,27^, while other studies neglected real-time operation^21–23,26^.

In^28^, the authors proposed a bi-level energy management system for the optimal real-time operation of grid-tied multi-nanogrids, aiming to efficiently coordinate distributed generation, storage, and load management. In^29^, a multi-level optimal energy management strategy for a grid-tied microgrid was developed, explicitly considering uncertainties in weather conditions and load demand to enhance operational reliability and cost efficiency. In^30^, an advanced microgrid optimization framework integrating price-elastic demand response with GRSO was proposed to improve both economic and environmental performance. However, the reviewed works generally lacked the adoption of efficient solution algorithms and a comprehensive statistical evaluation to rigorously validate the effectiveness and robustness of their proposed optimization and energy management approaches.

To address the nonlinear optimization challenges inherent in nanogrids, various solution techniques have been proposed. Classical deterministic approaches, particularly mixed-integer linear programming (MILP), have been widely employed in earlier studies to optimize both the design and operational scheduling of NGs^18,31,32^. Due to their reliance on specific data representations and linear approximations, these methods often face limitations when dealing with high-dimensional nonlinear optimization problems and may fail to guarantee global optimality. Consequently, numerous evolutionary and metaheuristic algorithms have been introduced to overcome these limitations. These include Particle Swarm Optimization (PSO)^7^, Artificial Bee Colony (ABC)^33^, and more recent approaches such as Aquila Optimizer (AO)^28^, Honey Badger Algorithm (HBA)^8^, and Genetic Algorithm (GA)^34^, which have demonstrated strong capability in efficiently handling nonlinearity, uncertainty, and complex search spaces.

As more powerful optimization algorithms are developed, greater cost savings and improved solution quality can be achieved. In this paper, the Artificial Gorilla Troops Optimizer (AGTO) is proposed. Table 1 summarizes the previous literature in comparison with the proposed work, clearly highlighting the methodological distinctions of the proposed bi-level AGTO-based EMS compared to recent state-of-the-art studies.

**Table 1:** Comparative literature survey to emphasis the novel contribution of this article.

Feature	Ref^4^	Ref^6^	Ref^7^	Ref^8^	Ref^16^	Ref^18^	Ref^21^	Ref^22^	Ref^28,29^	Proposed
Day-ahead scheduling	✓	✗	✓	✓	✗	✓	✗	✓	✓	✓
Real-time correction	✓	✗	✗	✓	✗	✗	✗	✗	✓	✓
Uncertainty (load + tariff + RES)	✗	limited	✗	limited	✗	✓	partial	partial	✓	✓
DSM integration	✓	✗	✓	✓	✓	✗	✓	✓	✓	✓
Statistical validation	✗	partial	✗	✗	limited	✓	✗	limited	✗	✓

Based on the previous literature review, the following research gaps are concluded. First, demand-side management is overlooked in several studies on the energy management of NGs. Second, some studies do not simultaneously consider the various sources of uncertainty in grid tariff, load, and atmospheric conditions that affect the renewable generations during real-time operation. Third, there is no rigorous statistical analysis across independent runs to evaluate the effectiveness of the proposed algorithm. To resolve these issues, this research introduces a bi-level energy management strategy that optimizes DERs scheduling to lower the daily operating costs of grid-connected NGs, and the AGTO is proposed to solve the resultant complex nonlinear problem efficiently, comparing its performance with HBA, AO, and PSO. The overall architecture of the proposed EMS is illustrated in Fig. 1.

**Fig. 1:**
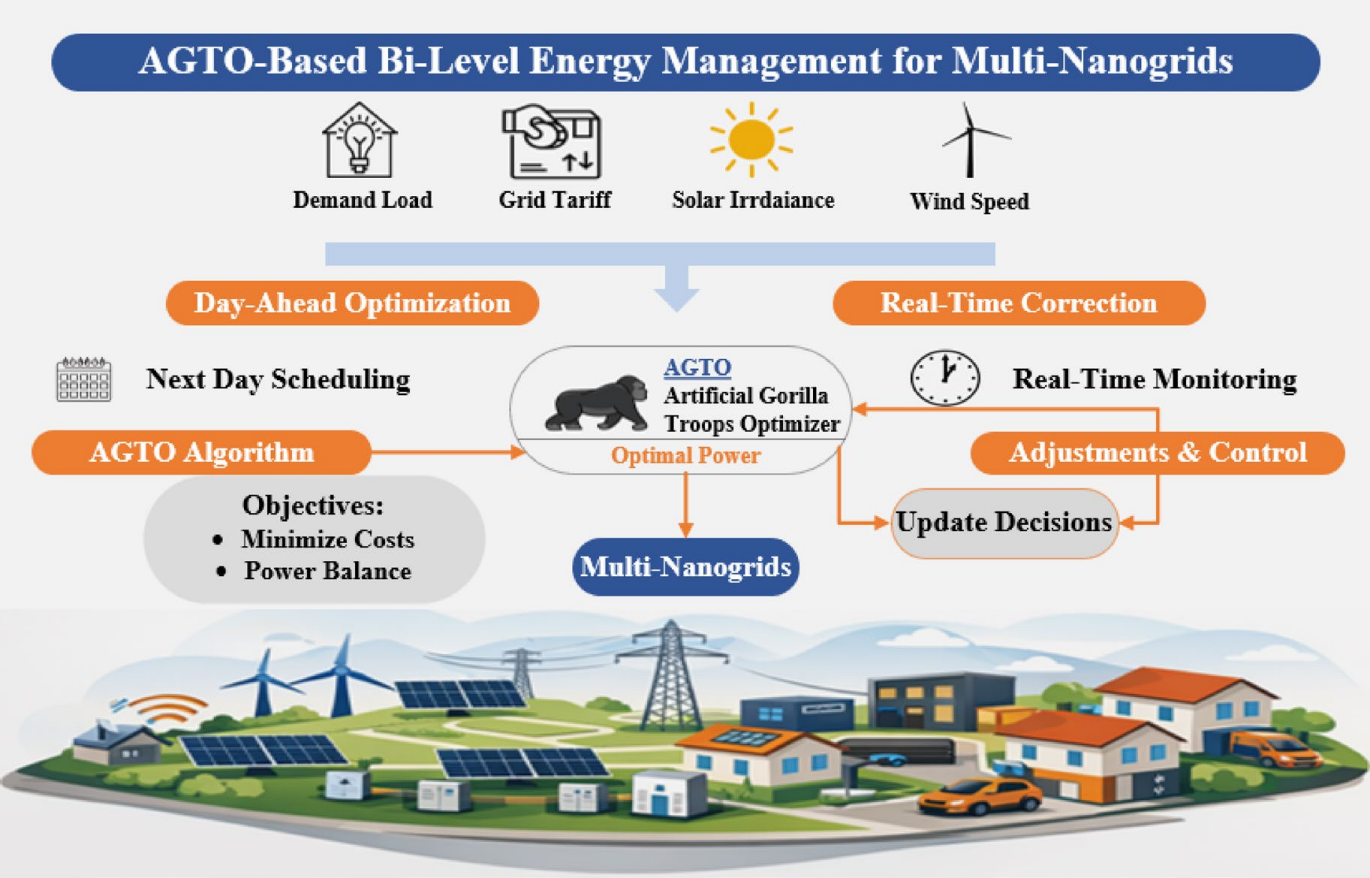
The overall framework of the proposed bi-level energy management system.

The principal contributions of this manuscript can be delineated as follows:

1. This work introduces a framework for the energy management of MNGs with the primary objective of minimizing daily operating cost while considering demand-side management using a load-shifting approach based on the day-ahead pricing curve.
2. To handle the uncertainties in weather conditions, grid prices, and load power, the output powers of the DERs within the NGs are dynamically rescheduled and adjusted in real time.
3. To mitigate the challenges posed by nonlinear optimization in the energy management problem of NGs, the AGTO is proposed, and a comparative analysis with PSO, AO, and HBA is conducted, accompanied by robust statistical validation, including multiple independent runs and Wilcoxon signed-rank testing, to verify convergence consistency and demonstrate comparative superiority.

The subsequent sections of this manuscript are as follows: the “System description” Section, which describes the proposed system. The “Proposed energy management system” Section illustrates the proposed EMS for achieving optimal daily operating cost. The optimization algorithm AGTO is discussed in the “Optimization techniques” Section. The “Simulation results” Section analyzes the simulation results across various scenarios. Finally, the last section presents the concluding remarks.

## System description

**Fig. 2:**
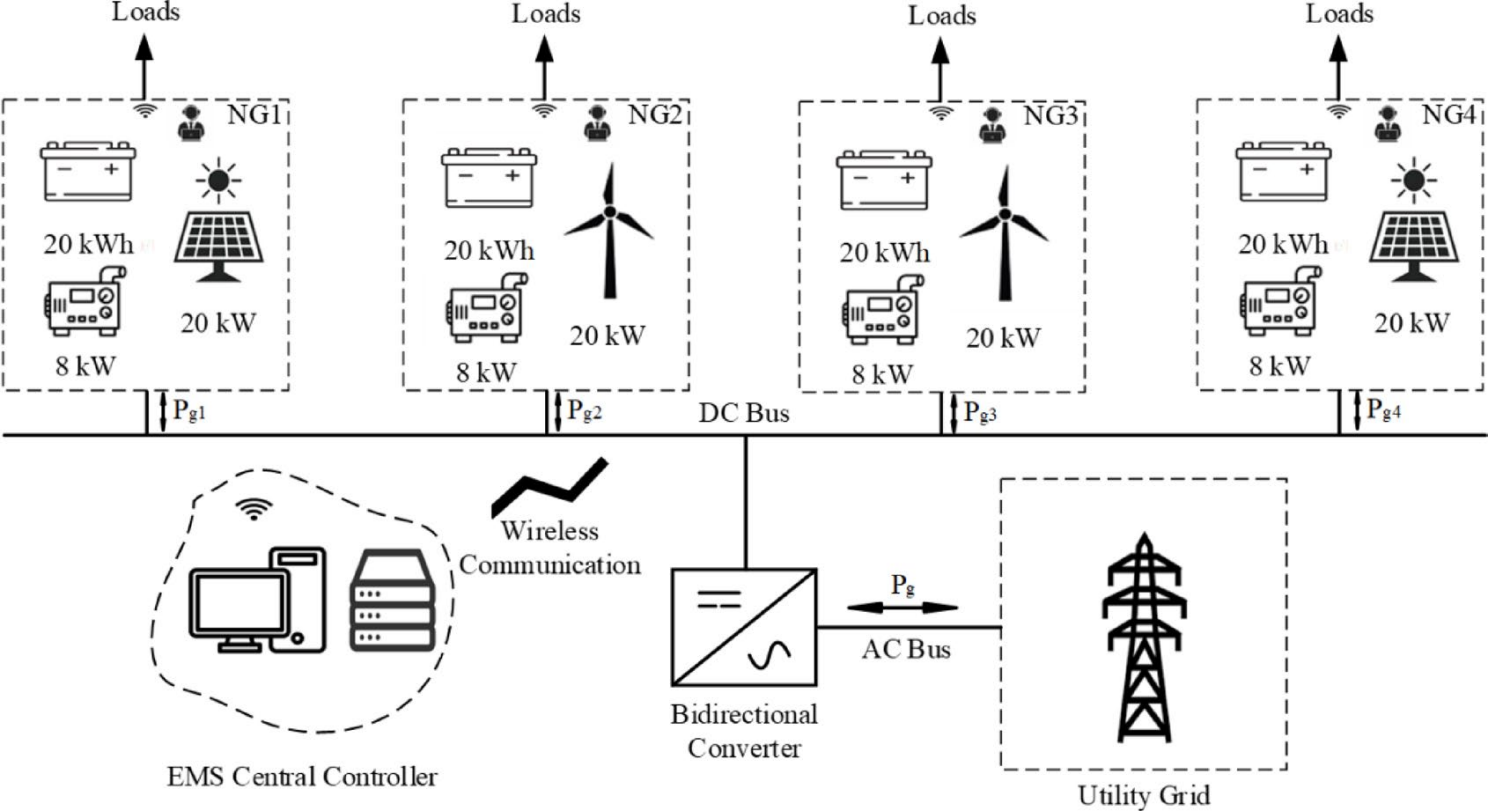
The proposed system.

The proposed system consists of four nanogrids connected through DC tie-lines and to the utility grid via an AC tie-line. NGs 1 and 4 contain five 4-kW PV modules, an 8-kW DG, and a 20-kWh battery. Conversely, NGs 2 and 3 comprise four 5-kW wind turbines, an 8-kW DG, and a 20-kWh battery, as depicted in Fig. 2. Since the distance is short, data from the NGs will be sent wirelessly via WIFI. Each distributed energy resource in the NG system operates with unique control modes and constraints. Battery storage alternates between charging and discharging states based on system requirements. Wind turbines and photovoltaic systems can function in either maximum power point tracking (MPPT) mode for optimal energy harvesting or power limiting mode during curtailment scenarios. Diesel generators operate within strict active power boundaries, maintaining generation between their minimum and maximum output limits. All DERs interface with the DC bus via converters and rectifiers. These power electronic interfaces enable precise control and regulation of the produced power from the NG system while ensuring optimal utilization of each DER’s capacity. The system parameters are shown below in Table 2^28^. All simulations were carried out using MATLAB R2020a on a workstation equipped with an Intel Core i5 processor (2.5 GHz) and 12 GB RAM.

**Table 2:** System parameters.

PV parameters					
$A = 20\;{\mathrm{m}}^{2}$	$\eta_{pv} = 18\%$	$\eta_{conv} = 94\%$	$N_{pv} = 5$	$P_{pv} \left( {\max } \right) = 4\;{\mathrm{kW}}$	
Wind turbine parameters					
$P_{w} = 5\;{\mathrm{kW}}$	$v_{ci} = 3\;{\mathrm{m}}/{\mathrm{s}}$	$v_{cf} = 25\;{\mathrm{m}}/{\mathrm{s}}$	$v_{r} = 10.5\;{\mathrm{m}}/{\mathrm{s}}$	$N_{w} = 4$	$\eta_{conv} = 92\%$
Diesel generator parameters					
$P_{dg} = 8\;{\mathrm{kW}}$	$a = 0.03\;\$ /{\mathrm{h}}$	$b = 0.2\;\$ /{\mathrm{kWh}}$	$c = 0.01\;\$ /{\mathrm{kW}}^{2} {\mathrm{h}}$	$P_{dg} \left( {{\mathrm{min}}} \right) = 1\;{\mathrm{kW}}$	$P_{dg} \left( {{\mathrm{max}}} \right) = 8\;{\mathrm{kW}}$
Battery parameters					
$B_{capacity} = 20\;{\mathrm{kWh}}$	$SOC_{min} = 0.2$	$SOC_{max} =$ 0.9	$SOC_{ini} =$ 0.5	$T = 1\;{\mathrm{h}}$	$\eta_{dis} = 0.9$
$\eta_{ch} = 0.9$	$N_{cycle} = 4000$	$CC_{Bat} = 300\;\$ / {\mathrm{kWh}}$	$C_{deg} = 0.00025\;\$ /{\mathrm{kW}}$	$B_{batt} \left( {{\mathrm{max}}} \right) = 18\;{\mathrm{kWh}}$	$B_{batt} \left( {\min } \right) = 4\;{\mathrm{kWh}}$

## Proposed energy management system

**Fig. 3:**
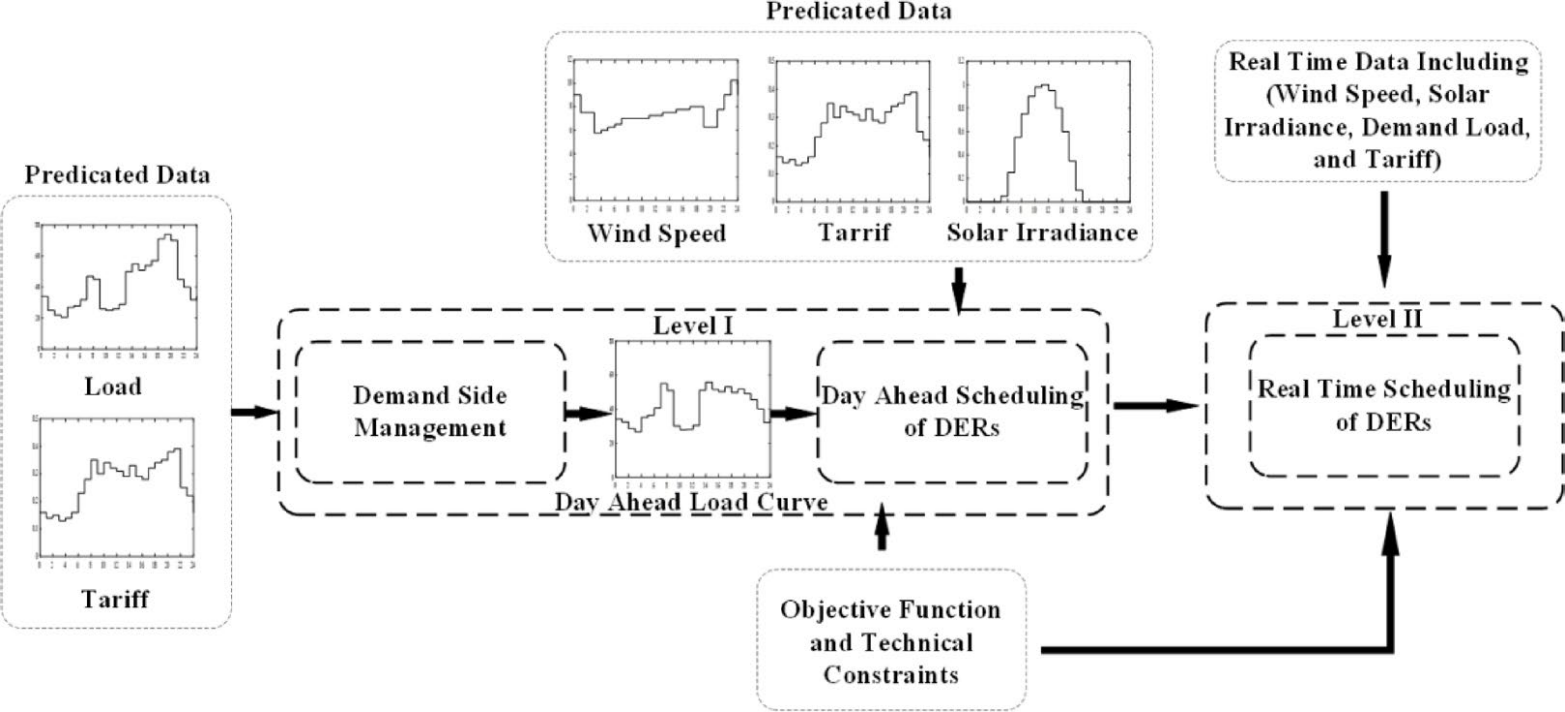
Flowchart of the proposed energy management system.

To reduce the daily operating cost, the recommended EMS is operated through day-ahead and real-time scheduling. The system utilizes predicated data (daily load curve, solar intensity, wind speed, and energy price) alongside real-time measurements to minimize operational cost while meeting demand. Key outputs include optimal generation schedules and power exchanged with the utility, as illustrated in Fig. 3. A real-time corrective control layer continuously compares actual vs. forecasted values. The energy tariff, wind speed, and solar intensity uncertainties in this work do not exceed 10%^29^. Due to inaccurate forecasting, the real demand load uncertainty may reach 20%^29^. When significant deviations occur due to forecasting errors, they trigger dynamic rescheduling of DERs and grid power exchange to maintain cost efficiency. The implementation of the proposed EMS occurs in two phases: firstly, there is the DSM using the load shifting technique with the predicted pricing curve; secondly, there is the regulation of power transmitted between NGs and the utility as well as power produced by DERs in NGs.

### Demand side management (DSM)

Electrical loads can be classified as either flexible or non-flexible. When it comes to scheduling, non-flexible loads like TVs, laptops, and microwaves are less flexible than flexible loads like electric water, washers, and heaters, which are examples of flexible loads. About 20–30% of all connected loads are managed. To minimize the cost of energy consumption, the day-ahead pricing curve can be used to move these loads from one time interval to another to implement DSM^35–38^.

### Determination of the optimal setpoints for DERs

This stage consists of two parts. In the first, the setpoints of sources for the day-ahead scheduling are established using previous data. The second involves real-time source setpoint adjustments to achieve cost-effective operation in the face of unpredictable load demand, weather, and energy pricing. The advanced metaheuristic algorithm, AGTO, is recommended to find the best operating points for batteries, diesel generators, and electrical power supplied with the utility; their effectiveness is contrasted with that of conventional optimization methods (PSO, AO, and HBA). To minimize the cost of energy consumption in the coming day, it determines the best time to charge and discharge batteries, the ideal setpoints for DGs, and when to buy and sell power from and to the grid. Alternatively, the Lagrange Multiplier approach is used to identify the best setpoints of DGs in NGs, which are then compared using the metaheuristic algorithm.

### Mathematical formulation

#### EMS objective function

The objective function is minimizing the total daily operating cost *C(t)* as Eq. (1):

1 $$C(t) = \sum {\left[ {FC(t) + C_{g} (t) + C_{B} (t)} \right]}$$

where *FC(t)* represents the diesel fuel cost, *C*_*g*_*(t)* indicates the grid cost, and *C*_*B*_*(t)* shows the operating cost of the battery, which are calculated as Eqs. (2)–(4)^8,29^:

2 $$FC(t) = a + b \times P_{dg} (t) + c \times P_{dg} (t)^{2}$$

3 $$C_{g} (t) = \lambda (t) \times P_{g} (t)$$

4 $$C_{B} (t) = \frac{{CC_{Bat} \times \Delta T \times P_{B,ch} (t) \times \eta_{ch} }}{{2N_{cycle} }} + \frac{{CC_{Bat} \times \Delta T \times P_{B,dis} (t) \times \eta_{ch} }}{{\eta_{ch} \times 2N_{cycle} }} + C_{\deg } \times \left| {P_{B} (t)} \right| + o\_m$$

where *a*, *b*, and *c* are fuel cost coefficients of the diesel, *λ* is the incremental fuel cost, *η*_*dis*_, *η*_*ch*_ is the battery discharging and charging efficiency, *CC*_*Bat*_ is the capital cost of the battery, *C*_*dg*,__*o_m*_ is the Degradation battery cost and the daily operating and maintenance cost, *N*_*cycle*_ is the number of battery life cycles, and *ΔT* is the sampling time (1 h).

### Constraints

The total power balance in the NGs is achieved as Eq. (5):

5 $$P_{g} (t) = P_{l} (t) - P_{pv} (t) - P_{wt} (t) - P_{dg} (t) - P_{B} (t), P_{g,\min } < P_{g} (t) < P_{g,\max }$$

where *P*_*pv*_ is the PV output power, *P*_*wt*_ is the power produced from the wind turbines, *d*_*dg*,_*P*_*B*_ is the power of the diesel generator and the battery, which are calculated as Eqs. (6)–(10)^28,29^:

6 $$P_{pv} = G \times A \times \eta_{pv} \times (1 - \alpha_{pv} (25 - T)) \times N_{pv} \times \eta_{conv}$$

7 $$P_{wt} = N_{w} \times \eta_{conv} \left\{ \begin{gathered} 0\quad \quad \quad \quad \quad \quad \quad \quad {\mathrm{if}}\;v_{cf} < v\;or\;v < v_{ci} \hfill \\ P_{w} \times \left( {\frac{{v^{3} - v_{ci}^{3} }}{{v_{r}^{3} - v_{ci}^{3} }}} \right)\;\;\,{\mathrm{if}}\;v_{ci} < v\;or\;v < v_{r} \hfill \\ P_{w} \quad \quad \quad \quad \quad \quad \quad \;{\mathrm{if}}\;v_{r} < v\;or\;v < v_{cf} \hfill \\ \end{gathered} \right.$$

8 $$P_{dg} (t) = \frac{\lambda (t) - b}{{2c}} , P_{dg,\min } < P_{dg} (t) < P_{dg,\max }$$

9 $$E(t) = \left\{ \begin{gathered} E(t - 1) - \frac{{\Delta T \times P_{B} (t)}}{{\eta_{dis} }}\quad \quad \;\;\;\,{\mathrm{if}}\;P_{B} (t) > 0 \hfill \\ E(t - 1) - \Delta T \times P_{B} (t) \times \eta_{ch} \;\;\,{\mathrm{if}}\;P_{B} (t) < 0 \hfill \\ \end{gathered} \right.$$

10 $$SOC(t) = \frac{{E(t)}}{{B_{{capacity}} }},SOC_{{\min }} < SOC(t) < SOC_{{\max }} ,P_{{B,\min }} < P_{B} (t) < P_{{B,\max }}$$

where *G* is the solar intensity, *A* is the PV module area, *η*_*pv*_, *η*_*conv*_ is the photovoltaic efficiency and the converter efficiency, *α*_*pv*_ is the Temperature coefficient of PV power, *T* is the ambient temperature, *N*_*pv*_ is the number of PV cells in the module, *P*_*w*_ is the rated power produced from one wind turbine, *N*_*w*_ is the number of wind turbines, *v*_*ci*_, *v*_*cf*_ is the cut-in and cut-off wind speed, *v*, *v*_*r*_ is the actual and rated wind speed, *B*_*capacity*_, *E* is the battery capacity and the stored energy in the battery, and *SOC* is the state of charge.

## Optimization techniques

Metaheuristic algorithms are often used to solve complicated optimization issues that are challenging for conventional approaches to solve. They are therefore employed in this study to find the best day-ahead battery and diesel generator setpoints at NGs. This study examines the performance of AGTO, HBA, AO, and PSO to identify the algorithm with the most effective search capability for finding the best value.

### Artificial gorilla troops optimizer (AGTO)

**Fig. 4:**
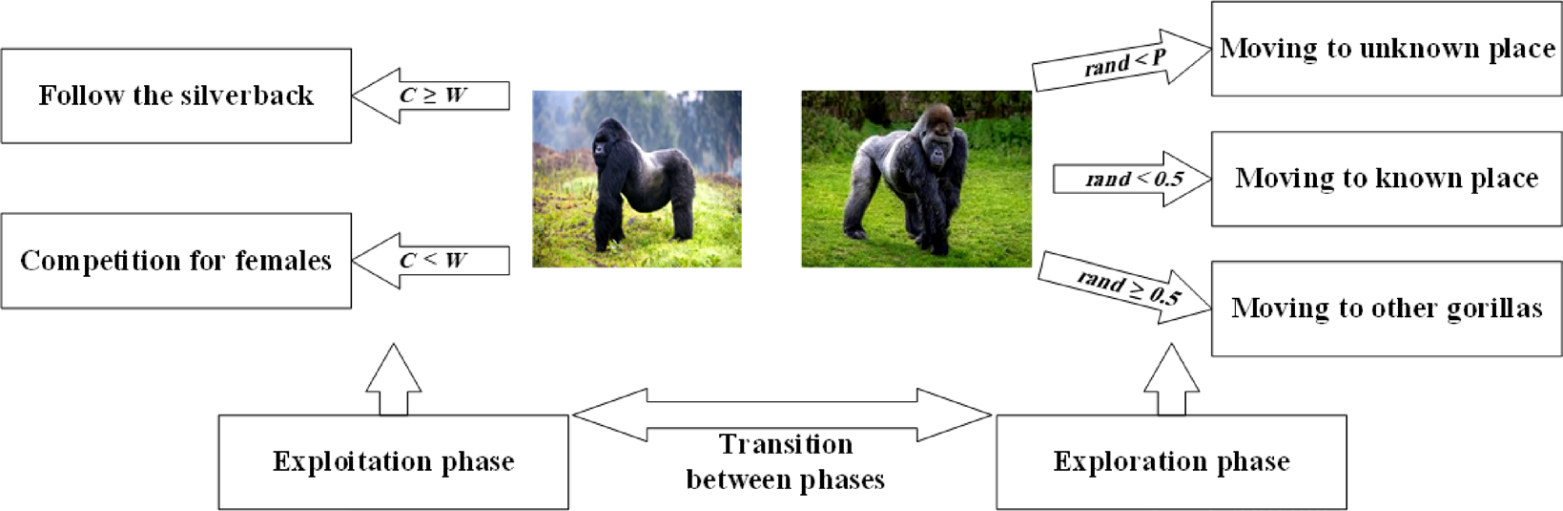
The main phases of the AGTO.

The AGTO is a swarm intelligence metaheuristic inspired by the collective foraging behavior of gorillas, which primarily seek food sources such as leaves, stems, and fruits. Gorillas exhibit a complex social structure, typically organized into cohesive groups known as troops. Each troop generally comprises a single dominant adult male, several adult females, and their offspring. As the largest extant primates, gorillas display sophisticated emotional responses and maintain strong familial bonds. The dominant adult male, identifiable by the silver-colored hair on his back, hence the term "silverback," assumes the role of group leader, undertaking responsibilities such as territory defense, decision-making, and directing troop members toward resource-rich areas^39^. The AGTO operates in two primary phases: exploration and exploitation. The exploration phase is modeled using three operators: traveling to an unknown location, approaching other gorillas, and moving toward a familiar location to simulate the dispersal and information-gathering behaviors observed in gorilla troops. In contrast, the exploitation phase employs two operators: tracking the silverback and competing for adult females to refine the search process and intensify convergence toward optimal solutions. Figure 4 provides a schematic representation of the main phases of the AGTO algorithm.

#### Exploration phase

Analysis of gorilla behavioral patterns reveals that gorillas typically live in the wild within groups led by a dominant silverback, whose authority is respected by all members. Occasionally, individuals separate from their original group. Upon dispersal, they may migrate to new natural habitats, which could be either familiar or unfamiliar to them. In the AGTO, each gorilla represents a candidate solution, while the silverback corresponds to the best candidate solution at a given stage of the optimization process. The position of each candidate gorilla, denoted as *G*_*X*_, is updated in every iteration according to Eq. (10)^39^. Prior to the optimization process, a random parameter *P* is initialized with a value [0, 1], which governs the migration behavior toward an unknown location. If *rand* < *P*, the gorilla is traveling to an unknown location. If *rand* ≥ *0.5*, the gorilla moves toward other gorillas. If *rand* < *0.5*, the gorilla instead moves toward a familiar location.

11 $$G_{X} (t + 1) = \left\{ \begin{gathered} LB + r_{1} \times (UB - LB),\quad \quad \quad \quad \quad \quad \quad \quad \quad \quad \quad \quad \quad \quad \quad \;\;rand < P \hfill \\ L + H + (r_{2} - C) \times X_{r} (t),\quad \quad \quad \quad \quad \quad \quad \quad \quad \quad \quad \quad \quad \quad \;rand \ge 0.5 \hfill \\ X(i) - L \times (L \times (X(t) - G_{r} (t)) + r_{3} \times (X(t) - G_{Xr} (t)),\quad rand < 0.5 \hfill \\ \end{gathered} \right.$$

The variables *rand, r*_*1*_*, r*_*2*_, and *r*_*3*_ are random variables [0, 1], where *LB* and *UB* show the lower and upper values for variables, and *G*_*X*_* (t* + *1)* is the position vector of the candidate gorilla, whereas *X(t)* shows the gorilla’s current location. The position vector of a randomly chosen gorilla is represented by *X*_*r*_ and *G*_*Xr*_. The following equations are used to determine *C*, *L*, and *H* as Eqs. (12)–(14)^39^:

12 $$C = (\cos (2 \times r_{4} ) + 1) \times \left( {1 - \frac{It}{{It_{\max } }}} \right)$$

13 $$L = C \times l$$

14 $$H = X(t) \times Z, Z = [ - C, C]$$

Hence, *It*_*max*_ and *It* represent the maximum number of iterations and the current iteration. *r*_*4*_ is a random value [0, 1], while *l* has a random number [-1, 1].

#### Exploitation phase

Two main behavioral strategies are used in the AGTO’s exploitation phase: following the silverback and competition for adult females. The silverback serves as the leader of the troop, making all strategic decisions, determining the group’s movement path, and directing members toward food sources. All gorillas in the troop comply with the silverback’s decisions, and he is also responsible for ensuring the safety and welfare of the group. However, as the silverback ages and weakens, a subordinate male, often referred to as a blackback, or other competing males may challenge his authority and attempt to assume leadership of the group. According to the *C*, which was determined using Eq. (12). In the event of *C* ≥ *W*, the gorillas follow the silverback; otherwise, adult females will complete.

#### Follow the silverback

Both the silverback and other troop members can perform their roles effectively during their younger years. For instance, male gorillas readily follow the silverback’s lead. Furthermore, everyone within the group has the potential to influence the behavior of other members. This social interaction can be mathematically modeled as follows Eq. (15)^39^:

15 $$G_{X} (t + 1) = X(t) + L \times M \times (X(T) - X_{Silverback} )$$

where: *X*_*Silverback*_ is the optimal solution, while *L* is calculated using the formula as Eq. (16)^39^:

16 $$M = \left( {\left| {\frac{1}{N}\sum\limits_{i = 1}^{N} {G_{Xi} (t)} } \right|^{g} } \right)^{\frac{1}{g}} ,g = 2^{L}$$

where: *N* and *G*_*Xi*_*(t)* is the number of gorillas and the position vector for each gorilla at iteration *t*.

#### Competition for adult females

A critical stage in the maturation of young male gorillas involves competing with other males for access to females. This competition is often intense, can persist for several days, and has noticeable effects on the behavior and dynamics of the entire troop.

17 $$G_{X} (i) = X_{Silverback} - (X_{Silverback} \times q - X_{t} \times q) \times A$$

18 $$q = 2 \times r_{5} - 1, A = \beta \times E$$

19 $$E = \left\{ \begin{gathered} N_{1} ,\quad \;rand \le 0.5 \hfill \\ N_{2},\quad\, rand > 0.5 \hfill \\ \end{gathered} \right.$$

**Fig. 5:**
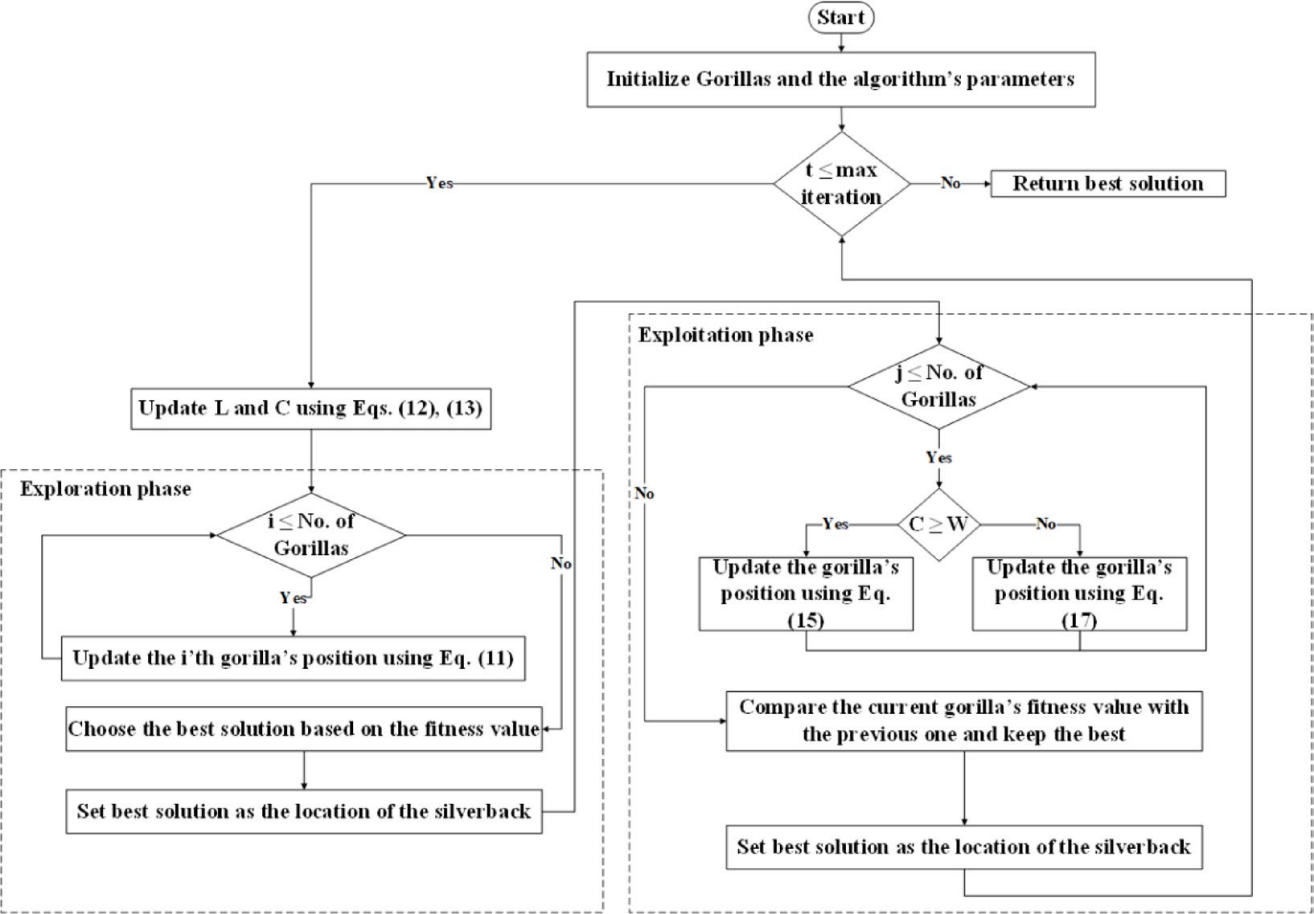
Flowchart of the AGTO algorithm.

In the AGTO algorithm, *q* represents the force impact, while *r*_5_ is a random value [0, 1]. Parameter *A* is a coefficient vector used to assess the intensity of conflict based on predefined variables *β* and *E*. The variable *E* models show how conflict severity influences the boundaries of candidate solutions. This is determined using a threshold of 0.5, when *E* ≥ *0.5*, its value is assigned from random values generated across the problem dimensions following a normal distribution; otherwise, *E* is assigned an arbitrary value within the normal distribution. In summary, Fig. 5 presents the flowchart of the AGTO algorithm.

### Proposed solution algorithm

Algorithm 1, provides the pseudocode for this technique, which solves the EMS problem by determining the optimum battery and diesel power setpoints that result in the minimum daily operating cost. In the current study, the conventional AGTO algorithm was employed as the main optimizer, and the stopping criterion was enhanced by combining the maximum number of iterations, convergence tolerance, and stagnation detection limit. The parameters of this algorithm are population size (120), max iterations (1500), tolerance value (10^−6^), number of runs (20), stagnation detection (20), exploration coefficient (3), and migration probability (0.03).

**Algorithm 1:**
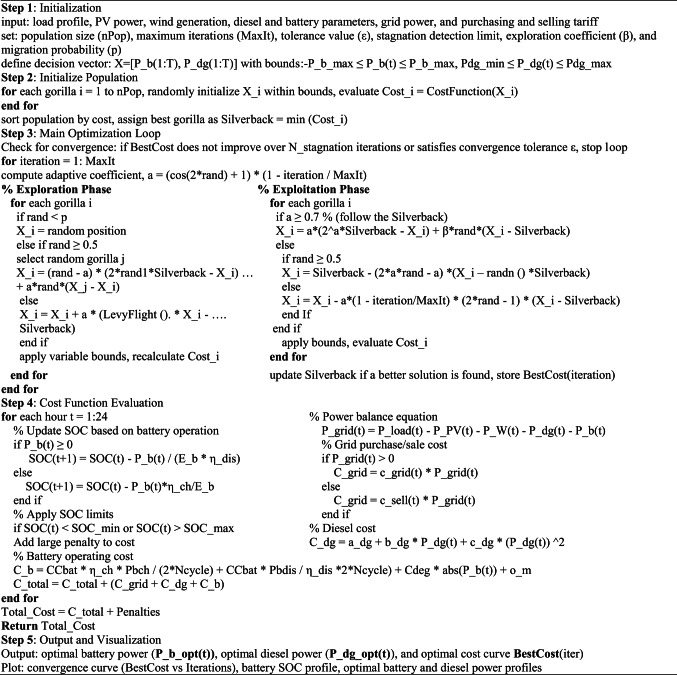
AGTO

## Simulation results

The results have been classified into two sections: the EMS of NGs based on day ahead scheduling and the real-time operation of grid-connected NGs.

### EMS of nanogrids based on day-ahead scheduling

**Fig. 6:**
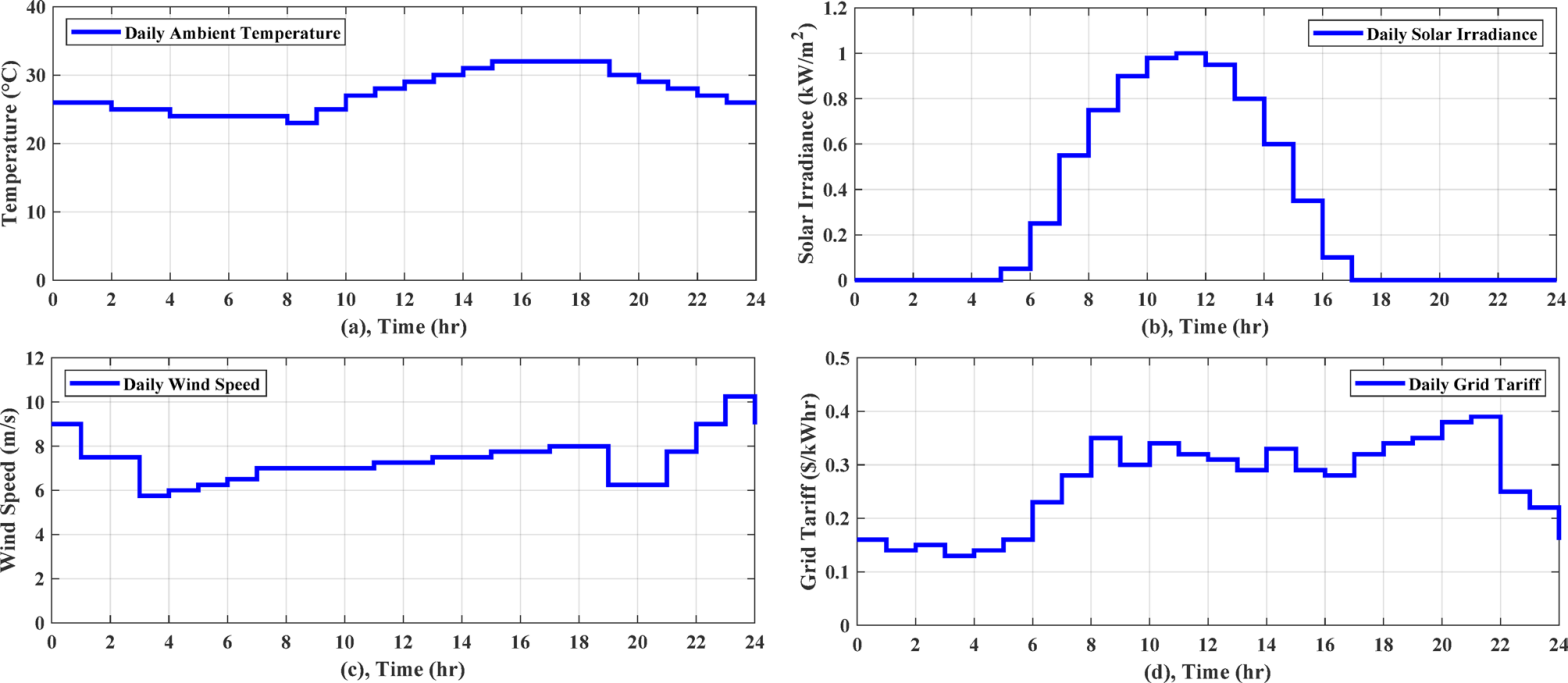
Forcasted daily ambient temperature, solar intensity, wind speed, and grid tariff^28^.

**Fig. 7:**
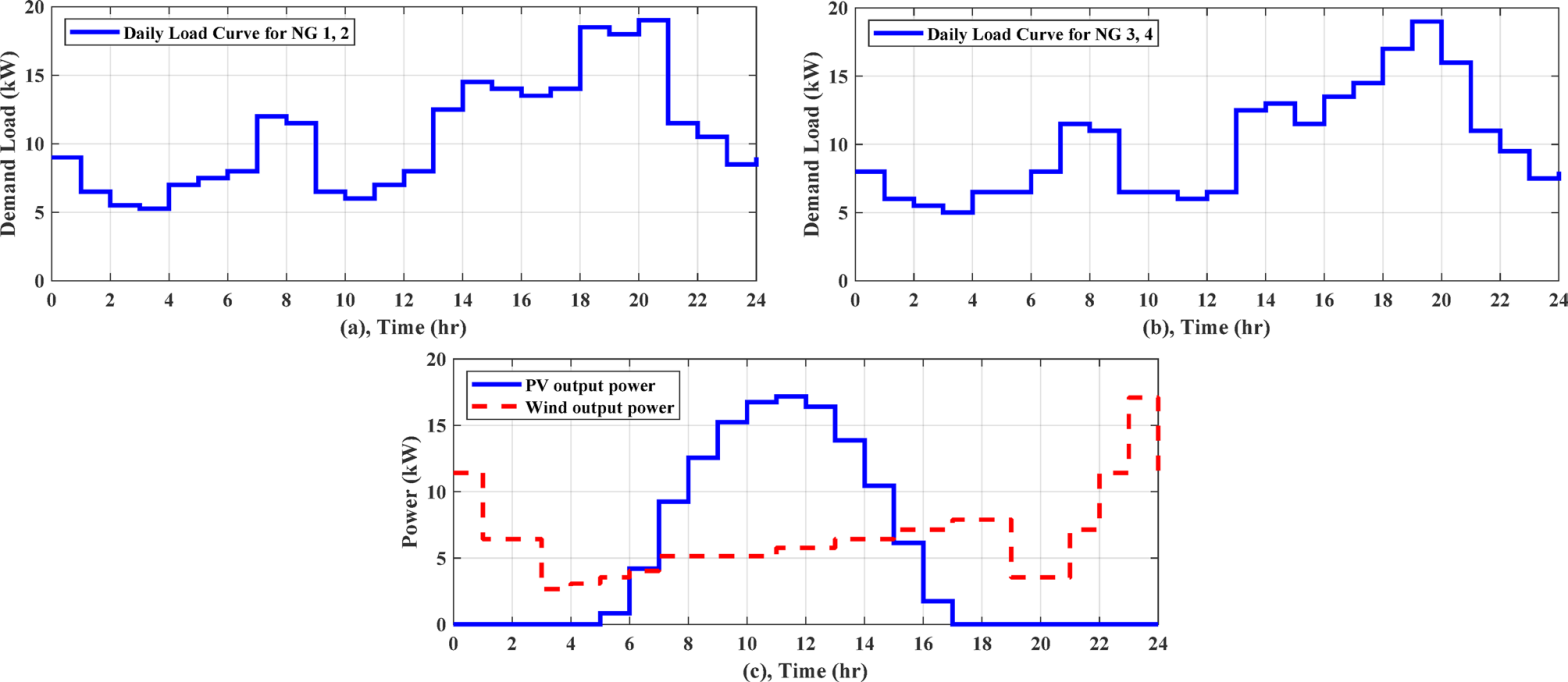
Power produced from solar, wind system, and load curve for NGs^28^.

For Zaafarana City, Egypt, the forecasted atmospheric temperature, solar intensity, wind speed, and grid tariff for the following day are displayed in Fig. 6. Figure 6b shows that at 11 am, the solar irradiation reaches its maximum value of 1 kW/m^2^ for the day. Consequently, as Fig. 7c illustrates the PV electricity varies according to the same schedule of solar irradiation. The wind speed fluctuates throughout the time and peaks about 11 pm, as depicted in Fig. 6c. The wind turbine’s power output follows the same shape of fluctuating wind speed, as observed in Fig. 7c. The daily load curve for NG 1 and NG 2 is presented in Fig. 7a, where the demand load fluctuates throughout the day, peaking at 19 kW at 8 pm and dropping to 5.25 kW at 3 am. The daily load demand for NG 3 and NG 4 is depicted in Fig. 7b, where the lowest load is 5.5 kW at 3 am and the maximum load is 19 kW at 7 pm. This paper describes a comparison of the performance of AGTO, HBA, AO, and PSO to identify the optimal optimization strategy that achieves the lowest daily operating cost.

#### Scenario 1: a single nanogrid operation

In this case, every NG works independently of the other surrounding grids.

- Case (I): In this study, the power of the diesel generator can be obtained once using the Lagrange Multiplier method and a metaheuristic technique to find the battery’s optimal power.

**Fig. 8:**
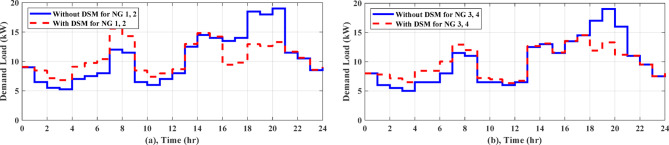
Daily load curve for NGs.

**Fig. 9:**
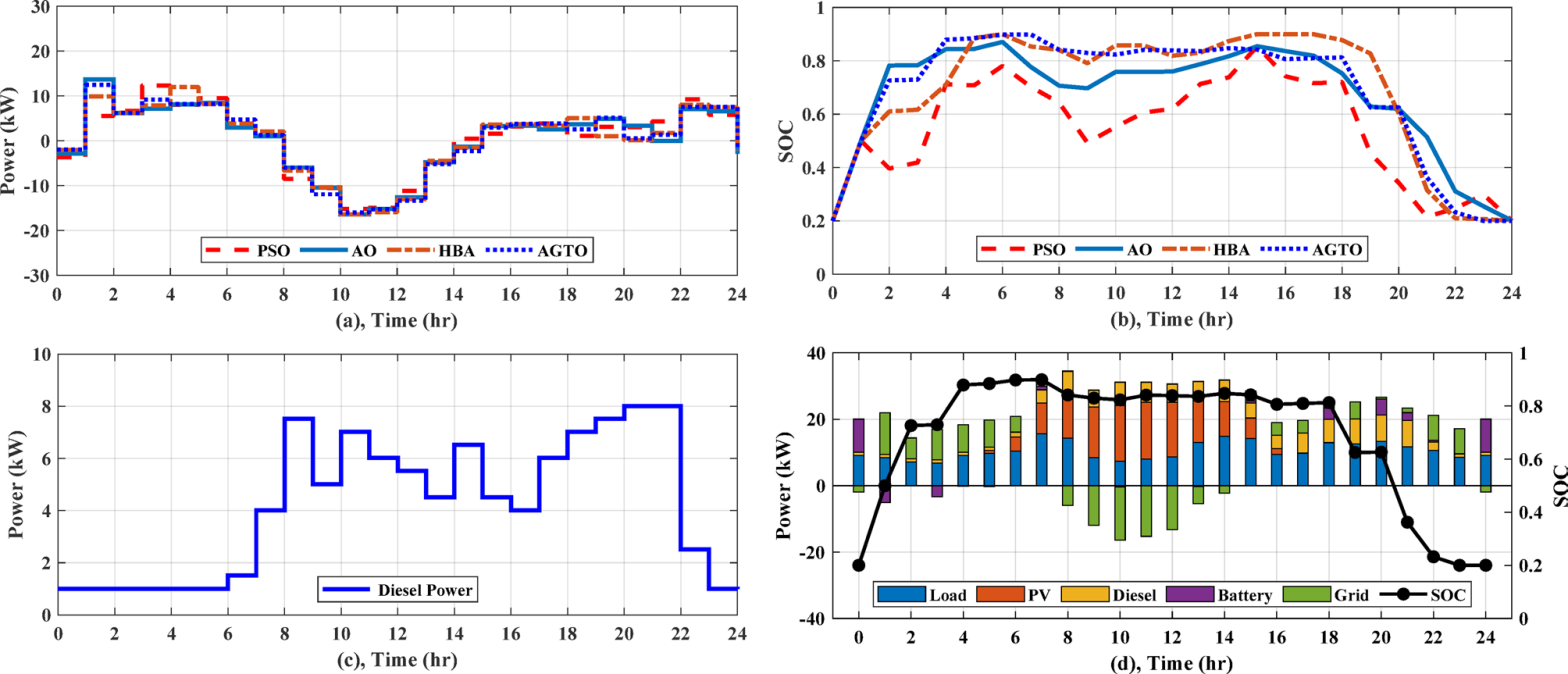
(**a**) Power exchange via grid, (**b**) Battery SOC, (**c)** Diesel power for any NG, and (**d**) Stacked power flow and SOC based on AGTO for NG1 during case (I).

In regard to NG1, without using DSM, AGTO, HBA, AO, and PSO achieved the daily operating costs of around $42.51, $43.02, $43.26, and $43.59, respectively. A DSM technique called load shifting with a day-ahead pricing profile is used to shift managed loads from times of high consumption to times of low generation and cost, which usually happen between 4 and 8 pm, as shown in Fig. 8a. As shown in Table 3, the implementation of DSM reduces peak load and improves the load factor. Consequently, the overall operating cost is reduced to $39.03, $39.06, $39.30, and $40.25 by AGTO, HBA, AO, and PSO, respectively. The power supplies from the utility to the NG1 at 8 am with about 9.15 kW for AGTO, 7.9 kW for HBA, 7.13 kW for AO, and 12.34 kW for PSO, as illustrated in Fig. 9a. Figure 9b indicates the battery’s state of charge, which at the last hour of the day achieves the minimum value of 20%. The power produced by the DG for each NG can be illustrated in Fig. 9c, where the amount of power produced fluctuates throughout the day in response to changes in energy prices, ultimately reaching the DG’s nominal output of 8 kW at 8 and 9 pm and the lowest value of 1 kW between 11 pm and 5 am.

**Table 3:** The comparison of load profiles before and after DSM.

	Before DSM	After DSM	Before DSM	After DSM	Before DSM	After DSM
	NGs (1,2)		NGs (3,4)		MNGs	
Peak Load (kW)	19.00	15.60	19.00	14.54	74.00	55.08
Peak load reduction (%)		17.89		23.47		24.74
Load factor	0.57	0.68	0.53	0.69	0.56	0.74

(b) Case (II): In this study, using a metaheuristic technique to obtain the powers of the DG and the battery.

**Fig. 10:**
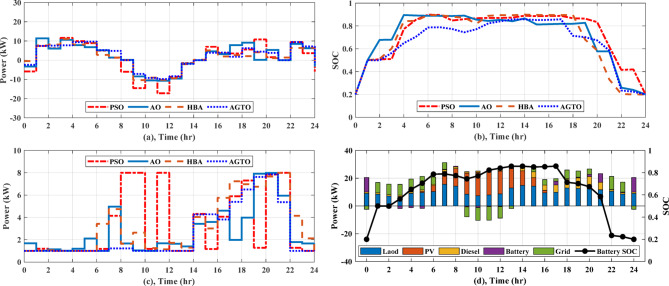
(**a**) Power exchange via grid, (**b**) Battery SOC, (**c**) Diesel power, and (**d**) Stacked power flow and SOC based on AGTO for NG1 during case (II).

**Fig. 11:**
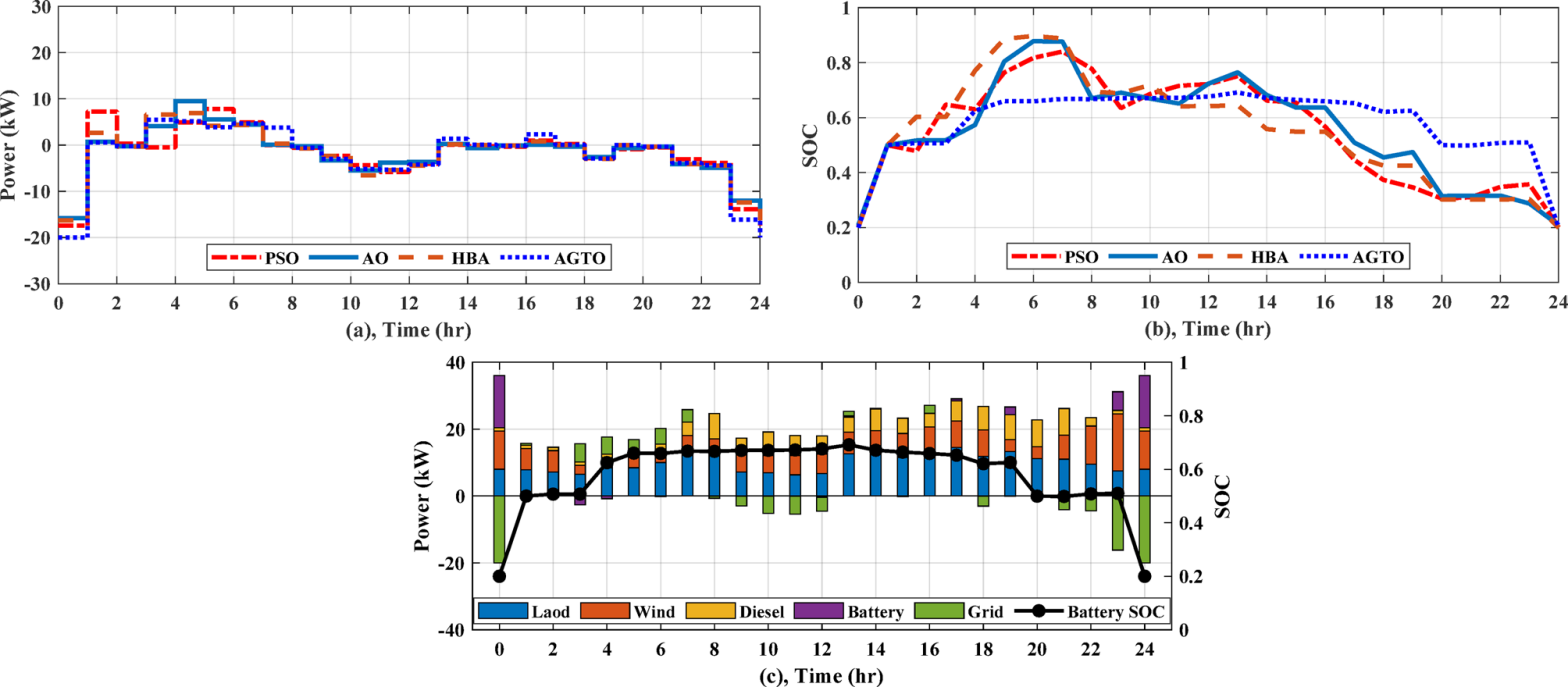
(**a**) Power exchange via grid, (**b**) Battery SOC, and (**c**) Stacked power flow and SOC based on AGTO for NG3 during case (I).

**Fig. 12:**
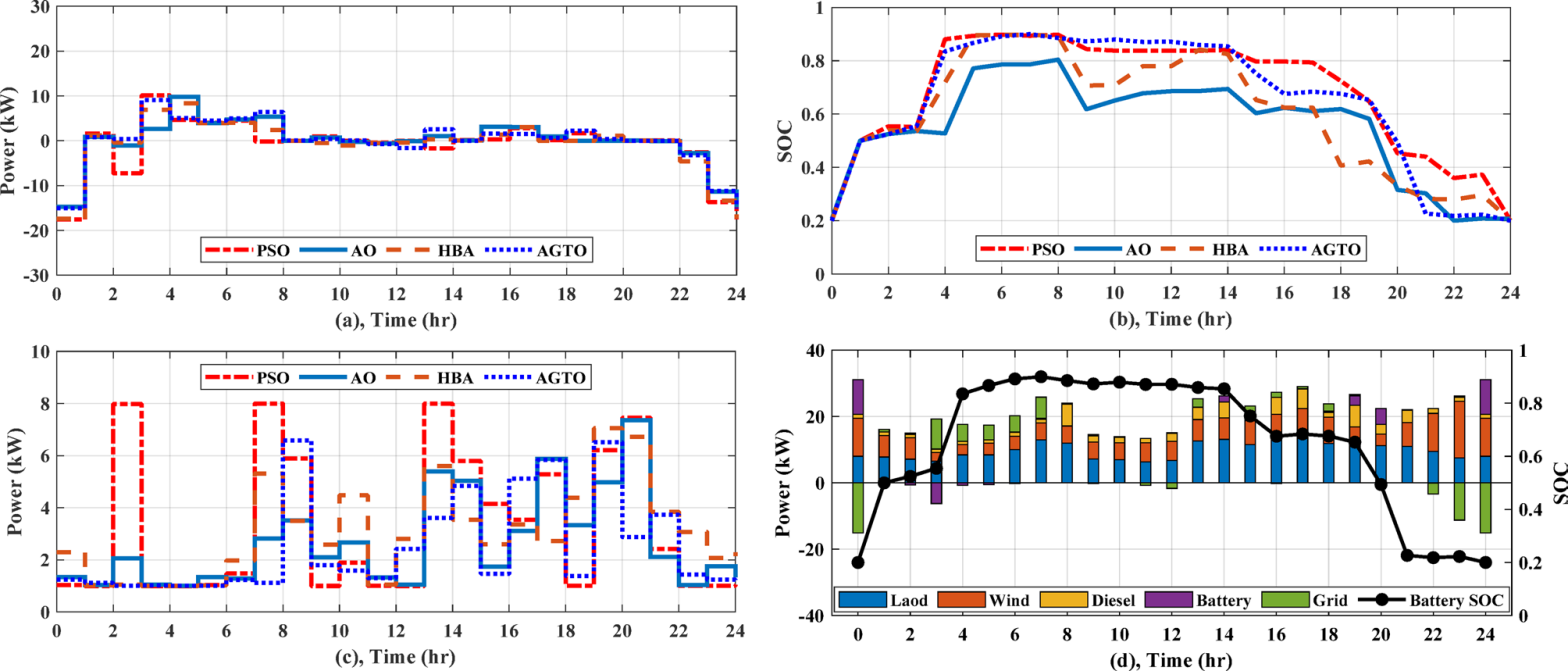
(**a**) Power exchange via grid, (**b**) Battery SOC, (**c**) Diesel power, and (**d**) Stacked power flow and SOC based on AGTO for NG3 during case (II).

As for NG1, the daily operating cost achieved by AGTO is about $40.34, while it is $40.40, $40.52, and $41.56 for HBA, and AO, PSO, respectively without applying DSM. With using DSM, the overall operating cost is decreased to $37.02 by AGTO, $37.16 by HBA, $37.29 by AO, and $38.75 by PSO. At 10 pm, power is transferred from NG1 to the utility with about 1.58 kW for PSO, 5.32 kW for AO, 1.07 kW for HBA, and 3.84 kW for AGTO, as indicated in Fig. 10a. The battery’s state of charge is shown in Fig. 10b, the battery is operated in the idle mode from 3 to 5 pm. The power produced by the DG can be indicated in Fig. 10c, the power at 5 pm is about 5.90 kW for PSO, 1.99 kW for AO, 7.23 kW for HBA, and 5.40 kW for AGTO. With respect to NG3, in the case (I), the daily operating cost achieved by AGTO is about $29.19, $29.36, $29.54, and $30.08 by HBA, AO, PSO, respectively, without DSM. Controllable loads are moved from peak to low periods, which usually take place between 6 and 8 pm, using DSM, as demonstrated in Fig. 8b. Consequently, the overall operating cost is reduced to $27.49 by AGTO, $27.76 by HBA, $28.10 by AO, and $28.45 by PSO. The power exchanged from NG3 to the grid at 10 am was about 4.38, 5.30, 6.56, and 5.15 kW for PSO, AO, HBA, and AGTO, respectively, as shown in Fig. 11a. Figure 11b indicates the SOC of the battery, the battery at 7 am is operated in discharging mode for PSO, AO, HBA, and idle mode for AGTO. In case (II), the daily operating cost is about $27.21, $27.44, $27.56, and $27.94 by AGTO, HBA, AO, and PSO, respectively, without DSM. With using DSM, the overall operating cost is decreased to $25.77 by AGTO, $25.79 by HBA, $25.92 by AO, and $26.36 by PSO. The power exchanged from the utility to the NG3 at 2 pm was about 0 kW, as seen in Fig. 12a. Figure 12b indicates the battery’s state of charge, the battery at 4 am is operated in charging mode for AO, HBA, and AGTO and the idle mode for PSO. The power produced by the DG at 1 pm was about 8, 5.39, 5.61, and 3.61 kW for PSO, AO, HBA, and AGTO, respectively, as observed in Fig. 12c.

To further verify the effectiveness and robustness of the proposed AGTO-based EMS, a comprehensive comparative analysis was conducted against other algorithms, as presented in Tables 4 and 5. The evaluation was performed over multiple independent runs using statistical indicators (mean, best, worst, and standard deviation). In addition, the Wilcoxon signed-rank test was employed to assess the statistical significance of the performance differences. The results confirm the superiority and stability of the proposed approach compared to the competing methods. Furthermore, as shown in Table 6, when compared to recent metaheuristic methods such as Logarithmic Mean-Based Optimization (LMBO)^40^, Dream Optimization Algorithm (DOA)^41^, and Goat Optimization Algorithm (GOA)^42^, AGTO achieves the lowest operational cost.

**Table 4:** Statistical results over 20 runs for NG1 with DSM.

Statistical results for NG1 over 20 runs	Daily operating cost ($) during Scenario 1, Case (I)				Daily operating cost ($) during Scenario 1, Case (II)			
	Best	Mean	Worst	Std	Best	Mean	Worst	Std
PSO	39.48	40.72	47.17	2.20	37.00	39.54	47.08	2.75
AO	38.98	39.45	40.45	0.47	37.16	38.09	39.15	0.60
HBA	39.08	39.42	40.79	0.40	36.76	37.15	37.96	0.36
AGTO	39.07	**39.36**	39.81	**0.19**	36.70	**37.07**	37.38	**0.20**

Significant values are in [bold].

**Table 5:** Wilcoxon Test over 20 runs for NG1 with DSM.

Wilcoxon test for NG1 over 20 runs	During Scenario 1, Case (I)		During Scenario 1, Case (II)	
	Wilcoxon p-value	Significance	Wilcoxon p-value	Significance
AGTO vs PSO	0.0001	Significant	0.0001	Significant
AGTO vs AO	0.8228	No Significant	0.0001	Significant
AGTO vs HBA	0.0001	Significant	0.7368	No Significant

**Table 6:** The daily operating cost for NG1 without DSM.

Algorithm	Daily operating cost ($)during Scenario 1, Case (I)	Daily operating cost ($) during Scenario 1, Case (II)
PSO	43.04	41.62
AO	42.95	40.48
HBA	42.72	40.37
**AGTO**	**42.41**	**40.09**
LMBO	44.76	43.41
DOA	45.77	40.91
GOA	42.63	40.12

Significant values are in [bold].

#### Scenario 2: operation of a grid-connected multi-nanogrids cluster

**Fig. 13:**
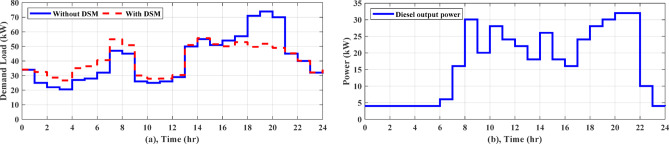
(**a**) Daily load curve for MNGs, (**b**) Diesel power for MNGs during case (I).

**Fig. 14:**
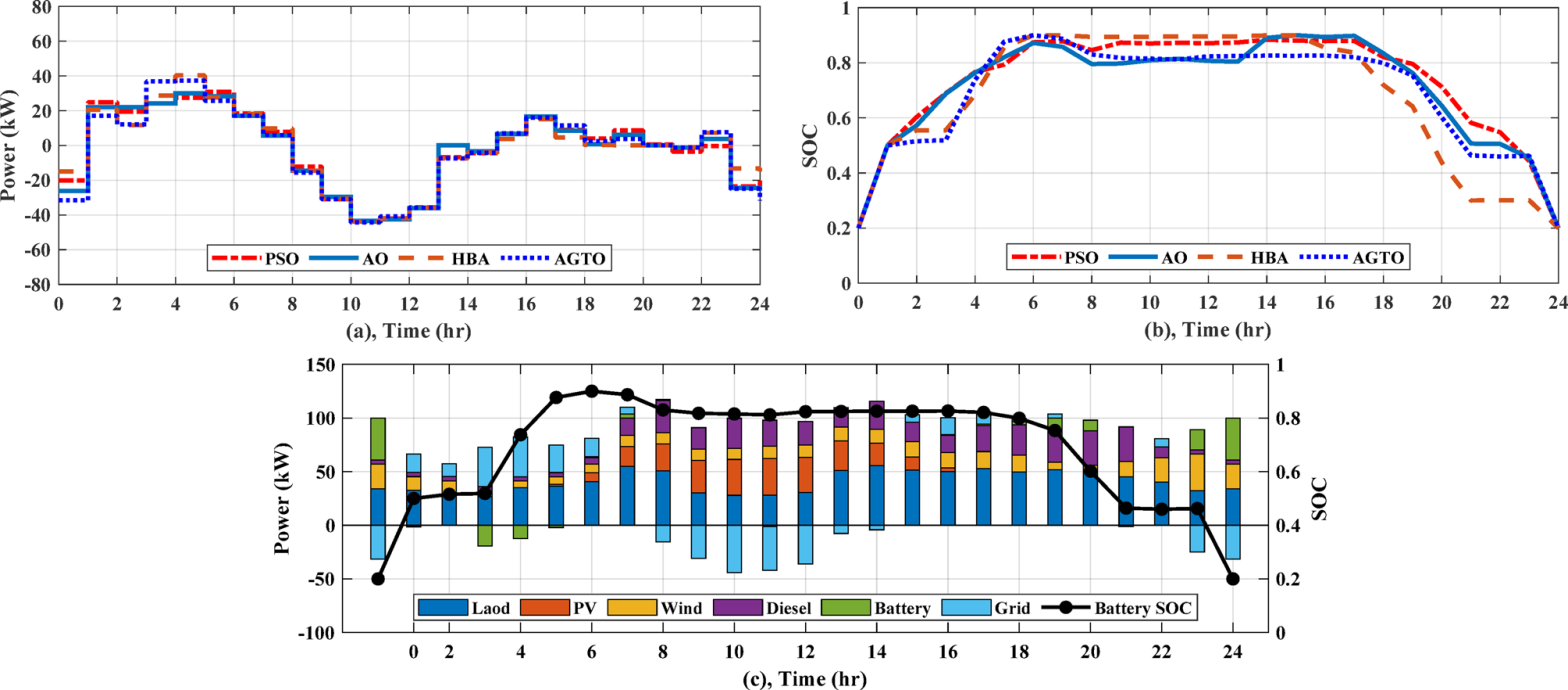
(**a**) Power exchange via grid, (**b**) Battery SOC, and (**c**) Stacked power flow and SOC for MNGs during case (I).

**Fig. 15:**
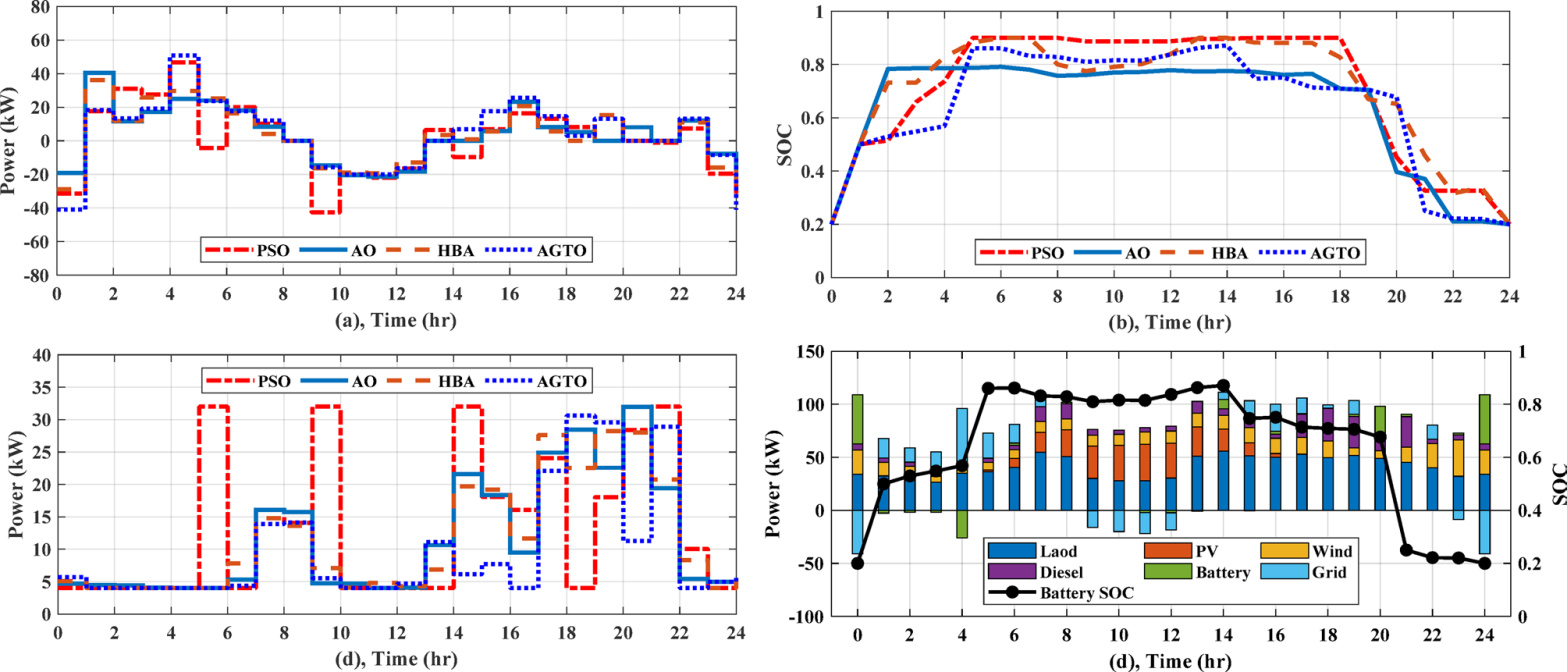
(**a**) Power exchange via grid, (**b**) Battery SOC, (**c**) Diesel power, and (**d**) Stacked power flow and SOC for MNGs during case (II).

In the present case study, four NGs working in a cluster are evaluated for their cooperation in working as a single microgrid when in grid-connected mode. In case (I), AGTO obtains a daily operating cost of around $132.73 for MNGs, whereas HBA, AO, and PSO provides $133.60, $134.26, and $135.56 with DSM, respectively. Figure 13a shows the daily demand load, the peak demand reaching to 54.93 kW at 7 am and off-peak demand reaching to 26.65 kW at 3 am. Figure 14a displays the power transfer to the utility from 8 am to 2 pm (-ve power). Figure 14b illustrates the SOC of the battery, which shows that it is charged for a certain period of time, discharged for another period, and then idle mode for another time. The power generated by the DGs of the NGs is displayed in Fig. 13b, with a minimum value of 4 kW from 11 pm to 5 am and a maximum value of 32 kW at 8 and 9 pm. In case (II), AGTO’s daily operating cost is around $123.72, but HBA, AO, and PSO provides $124.60, $125.35, and $128.42, respectively. Figure 15a shows the power transfers to and from the utility, which indicates that it is exchanged from NGs to the utility for a certain period of time and otherwise for another time. Figure 15b illustrates the battery’s state of charge, which shows the different modes of the battery. The power produced by the NGs’ diesel generators is displayed in Fig. 15d.

Compared to the base scenario1, case (I) without DSM, the day-ahead energy consumption cost drops by roughly $14.25 by AGTO, $13.38 by HBA, $12.72 by AO, and $11.42 by PSO, while in case (II), AGTO, HBA, AO, and PSO reduce the daily operating cost to roughly $23.26, $22.38, $21.63, and $18.56, respectively.

The AGTO algorithm to get the optimal setpoints of the diesel generator and the battery with the DSM technique, when considering the daily operational cost, performs better than the HBA, AO, and PSO algorithms, with a cost-saving percentage of about 15.83%.

#### EMS of grid-connected multi-nanogrids based on real time operation

**Fig. 16:**
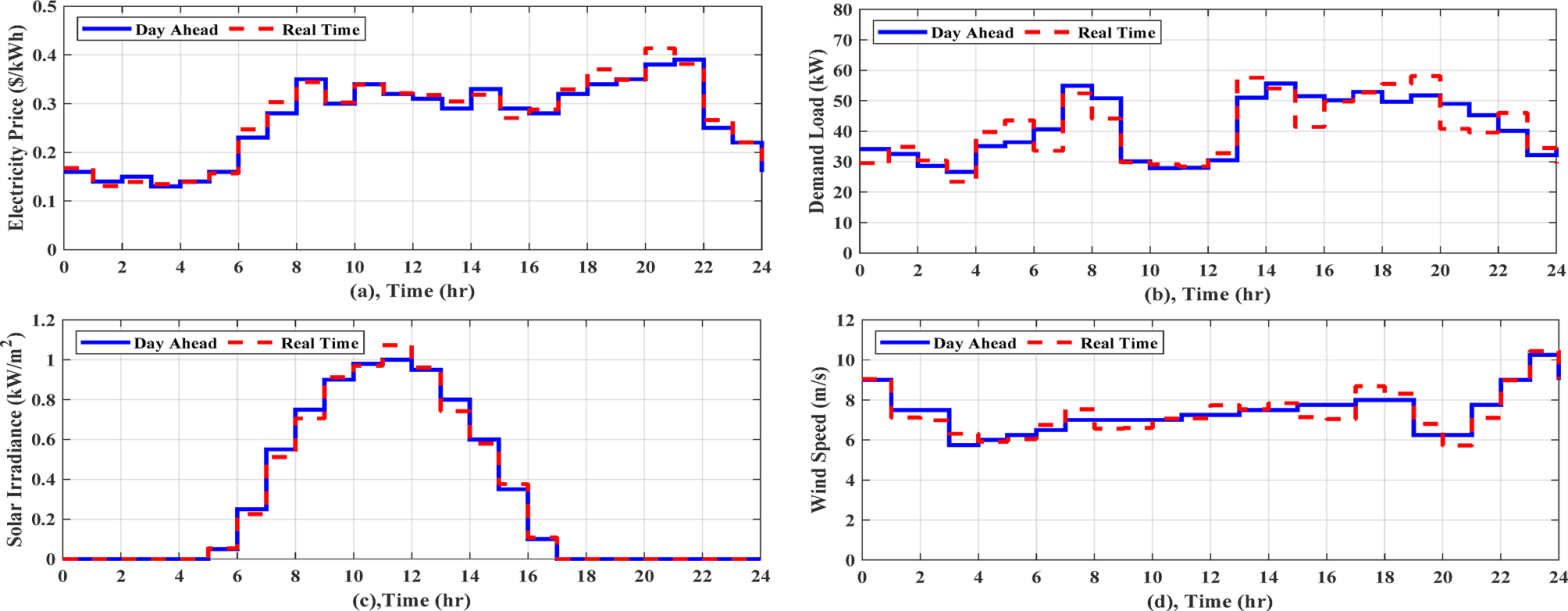
Forcasted and real grid tariff daily, demand load, solar intensity, and wind speed.

**Fig. 17:**
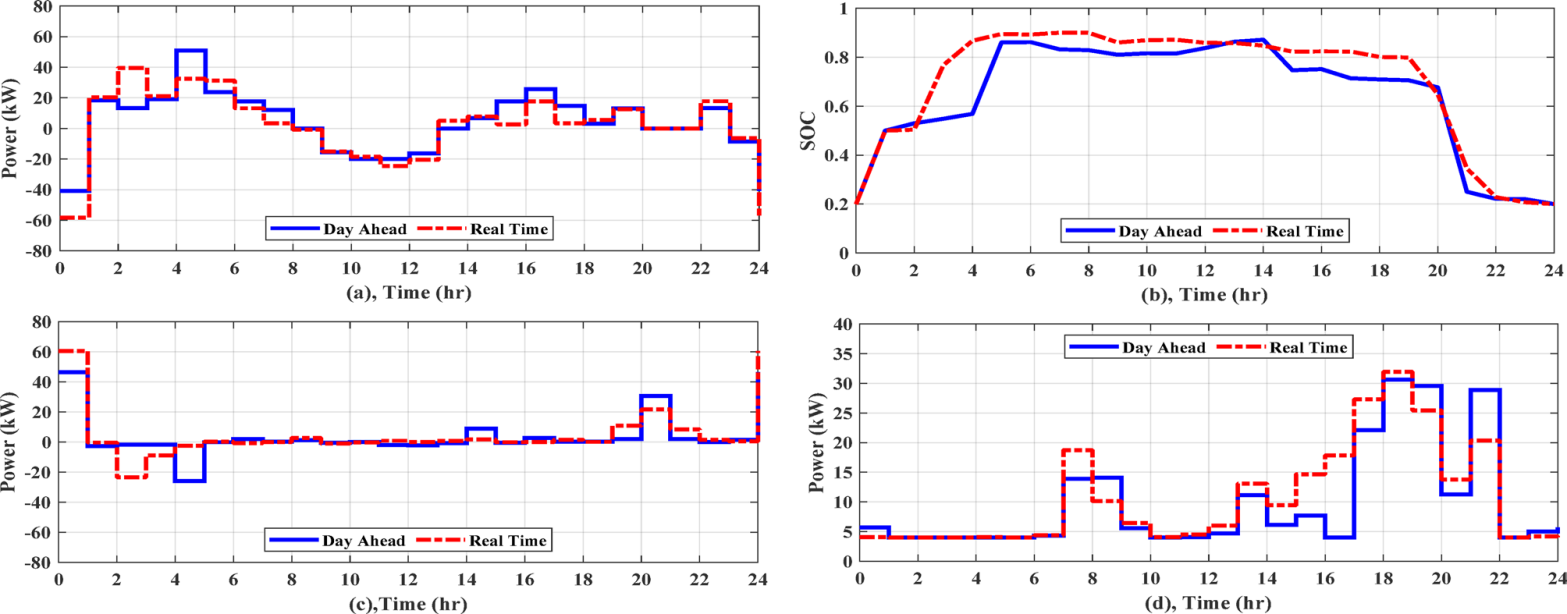
(**a**) Power exchange via grid, (**b**) Battery SOC, (**c**) Battery power, (**d**) Diesel power for MNGs.

Forecasts of weather conditions and electrical loads are always subject to a percentage error. In order to ensure economical operation under expected uncertainties, such as load demand, weather, and grid tariff, real-time EMS updates and reschedules the setpoints of sources determined from day-ahead scheduling. In this study the forecasted data obtained by AGTO from scenario 2 and case (II) are used to compare with real-time data. Considering the uncertainty of solar intensity, wind speed, and grid pricing is less than 10%, the actual load variability may reach 20% due to inaccurate predictions^28,29^. Regarding communication delays and data latency, the framework assumes that typical communication delays (in the order of milliseconds to a few seconds in modern wired or wireless smart grid communication infrastructures) are significantly shorter than the 1-h rescheduling interval. Therefore, such delays do not affect the feasibility or stability of the scheduling decisions. As illustrated in Fig. 16a, the real electricity price may fluctuate by a few percentage points from the predicted price, and the actual load and the predicted load differ significantly, as demonstrated in Fig. 16b. The solar intensity and wind speed statistics in real time are shown in Fig. 16c and d. Figure 17a shows how power is transferred to and from the utility over the day. Figure 17b illustrates the battery’s state of charge, which at the last hour of the day achieves the minimum value of 20% for both day-ahead and real-time scheduling, and Fig. 17c displays the battery’s output power, which operates in all modes over the day. The power provided by the DGs of the NGs is shown in Fig. 17d. The real-time EMS saves $5.24 per day; the daily operating cost decreases by approximately 4.24%, from $123.72 to $118.47. The daily operational cost summary based on simulation results will be reviewed in Table 7.

**Table 7:** The summary of the daily operating cost.

	Without load management				With load management				Without load management				With load management			
	PSO	AO	HBA	AGTO	PSO	AO	HBA	AGTO	PSO	AO	HBA	AGTO	PSO	AO	HBA	AGTO
Daily operating cost ($) during Scenario 1, Case (I)									Daily operating cost ($) during Scenario 1, Case (II)							
NG1	43.59	43.26	43.02	42.51	40.25	39.30	39.09	39.03	41.56	40.52	40.40	40.34	38.75	37.29	37.16	37.02
NG2	34.38	33.79	33.35	33.25	31.52	31.41	31.04	30.76	32.85	31.83	31.74	31.46	30.54	29.66	29.16	28.91
NG3	30.08	29.54	29.36	29.19	28.45	28.10	27.76	27.49	27.94	27.56	27.44	27.21	26.36	25.92	25.79	25.77
NG4	38.93	38.91	38.65	38.61	36.98	36.69	36.51	36.07	36.99	36.48	36.19	36.06	34.50	33.70	33.60	33.28
Total	**146.98**	145.50	144.38	143.58	137.20	135.50	134.40	133.41	139.34	136.39	135.77	135.07	130.15	126.57	125.71	124.98
Daily operating cost ($) during Scenario 2, Case (I)									Daily operating cost ($) during Scenario 2, Case (II)							
MNGs	144.91	144.43	143.47	142.04	135.56	134.26	133.60	132.73	138.08	135.78	134.55	133.67	128.42	125.35	124.60	**123.72**
Day-ahead based EMS using AGTO, Case (II)									Real time-based EMS using AGTO, Case (II)							
MNGs								**123.72**								**118.47**

Significant values are in [bold].

## Conclusion

To lower daily operating costs while fulfilling the technical constraints, an effective EMS is proposed. It performs load management and determines the optimal setpoints for distributed energy resources via day-ahead and real-time scheduling within multi-connected nanogrids. The advanced metaheuristic algorithm, AGTO, is employed to identify the optimal setpoints of both the battery and the diesel generator. Its performance is compared with that of the HBA, AO, and PSO. The proposed load management strategy, based on load shifting under an expected pricing curve, reduces operating costs by approximately 7.44% by shifting controllable loads from high to low-cost periods. When four NGs operate collaboratively with managed loads, the total daily energy consumption cost is reduced by roughly 8.40% compared to the case where each NG operates independently without DSM. For the following day’s operation, the results obtained by the proposed AGTO-based algorithm outperform those achieved by HBA, AO, and PSO. Due to fluctuations in predicted data, AGTO is integrated with the real-time EMS to identify updated setpoints for DERs, batteries, and diesel generators in the multi-connected NGs. This approach yields additional daily cost savings of approximately 4.24% compared to day-ahead scheduling. Considering technical constraints and uncertainties, the proposed EMS for multi-connected NGs effectively maintains system balance in a cost-efficient manner.

Future work will focus on extending the proposed AGTO-based EMS framework to incorporate additional practical considerations. In particular, the model can be expanded to integrate Electric Vehicles (EVs) as flexible loads or distributed storage resources and to include more advanced demand-side management strategies. Moreover, large-scale implementations with higher numbers of interconnected nanogrids will be investigated to address computational efficiency and scalability aspects. A comprehensive annual economic assessment under seasonal operating scenarios will also be conducted to provide a more realistic evaluation of long-term technical and economic benefits, and a detailed delay sensitivity analysis is considered a valuable extension and will be addressed.

## Data Availability

The datasets used and/or analysed during the current study are available from the corresponding author on reasonable request.
